# An Exploratory Study into the Backgrounds and Perspectives of Equine-Assisted Service Practitioners

**DOI:** 10.3390/ani14020347

**Published:** 2024-01-22

**Authors:** Rita Seery, Deborah Wells

**Affiliations:** Animal Behaviour Centre, School of Psychology, Queen’s University Belfast, Belfast BT7 1NN, UK; d.wells@qub.ac.uk

**Keywords:** animal-assisted therapy, equine-assisted services, horses, practitioners

## Abstract

**Simple Summary:**

Horses are being increasingly incorporated into health and wellbeing treatments and interventions. These Equine-Assisted Services (EASs) vary widely in both theoretical and practical applications. However, until now, the experiences and perspectives of the practitioners of these services have received little attention. To address this gap in knowledge, EAS practitioners were asked to complete a survey exploring the service they provide, practice patterns, background education, perceived knowledge, challenges faced and issues moving forwards. Practitioners’ backgrounds were found to have a significant influence on both the type of EAS provided and respondents’ perceived knowledge. Most practitioners received training specific to the service they provided, with block release being a common trend. Just under half the sample had received under 20 days of training. Horse-specific training was relatively uncommon. Practitioners reported client and horse welfare, financial sustainability and raising awareness as the most important challenges facing their service. This study highlights the diversity within the field of EAS and the challenges faced by practitioners, as well as possible opportunities for growth. More progress is needed to support practitioners in order to grow, professionalise and legitimise these services.

**Abstract:**

Equine-Assisted Services (EASs) are commonplace in today’s society, but vary widely in both theoretical and practical applications. Until now, practitioners’ experiences and perspectives in relation to these services have received little attention. To address this, a purpose-designed online questionnaire was distributed to EAS practitioners, exploring issues relating to the nature of the service provided, practice patterns, practitioner education, perceived knowledge, challenges faced and the future direction of these services. An analysis revealed a significant association between practitioners’ backgrounds and the nature of the service offered, as well as perceived knowledge. Median EAS training received to first practice was 20 days of block release over a year; however, nearly half of the sample (42.4%) reported less training than this. Equine-specific training was more limited, with 41.5% of practitioners having no horse-relevant qualifications. The most important challenges reported by practitioners involved client and equine welfare, financial sustainability and raising awareness of EAS. This research highlights the diverse nature of EAS and also raises important challenges and possible opportunities for development. Findings suggest that more progress is needed to professionalise and legitimise the area to support and help practitioners provide the best service for all concerned.

## 1. Introduction

The domestic horse has long been recognized for its therapeutic benefits. Even prior to Hippocrates formally describing the therapeutic effects of riding, horses had been regarded as helpful for rehabilitating soldiers in ancient Greece for several centuries [1,2]. The last 70 years has seen the popularity and provision of horse-related activities for human-related health dramatically increase, with a number of therapeutic riding organisations (e.g., Riding for the Disabled, UK, and the Professional Association of Therapeutic Riding, USA) established around the globe to foster developments in this area. Research exploring the efficacy of these riding programmes has reported physical health benefits for people suffering from a wide range of conditions, including cerebral palsy [3,4], multiple sclerosis [5], speech delays [6] and various other disabilities (for a review, see [7]). In addition to these mounted activities, the last 40 years has witnessed a move towards incorporating horses in different ways, primarily in an effort to improve people’s mental health but also to enhance learning and skills development. These more recent programmes, which can be ground-based, mounted, or mixed in nature, are diverse in terms of the services provided, practitioner competencies, target outcomes and populations catered for. The research conducted in this area is also variable, although it tends to focus largely on the merits of such programmes for people with specific conditions, e.g., Autism Spectrum Disorder [8,9,10], Attention Deficit Hyperactivity Disorder [11], PTSD [12], at-risk youth [13], and groups with a mental or emotional health outcome focus [14].

Whilst the above studies show encouraging results, methodological weaknesses are endemic to this field of study, with problems including a lack of randomized control trials [15], small sample sizes [16], a paucity of detail regarding the animals incorporated and the nature/delivery of the programme itself (for a review, see [17]). The field also presents other problems. For example, Equine-Assisted Services (EASs) consist of a spectrum of approaches, with the inclusion of horses as a unifying thread. This richness in the diversity of approaches, whilst a potential positive, has led to confusion in terminology, with generic terms, such as “equine therapy” or “horse therapy”, as well as the improper use of terms such as “hippotherapy”, being commonplace. The lack of uniform terminology has, until now, provided the public and researchers with little information as to the services on offer, the clientele targeted or the competencies of the practitioners involved. In an effort to address these issues, Woods and colleagues [18] recommended the adoption of optimal terminology (with EAS as the new umbrella term), dividing the field into three areas: Equine-Assisted Therapy, in which horses are incorporated into an established mental or physical health practice; Equine-Assisted Learning, where horses are integrated into a coaching or educational service; and Horsemanship, in which learning about, and taking part in, horsemanship activities is conducted, with enhancements in human health and wellbeing as the target outcomes. The emphasis on naming a human therapy or service with a mostly “equine-assisted” root has done much to provide clarification, especially amongst the research community, but remains a work in progress in terms of the universal standardisation of terminology [19].

A lack of standardisation and clarification within EAS is not limited solely to terminology. Although some international guidelines (e.g., the HETI Ethical Guidelines and IAHAIO white paper) are available, there is no independent body for the field to oversee or govern standards in terms of the services provided, practitioners’ experiences, ethical frameworks, or guidelines for best practices in terms of horse or client welfare specific to EAS. Indeed, to our knowledge, there are few situations in which minimum education or competency standards exist for the provision of any EAS beyond what is required for mental or physical health professional competencies. In addition, practitioner perspectives, practice patterns, backgrounds, educational/training status, client base and perceived benefits and challenges have been poorly studied across the EAS spectrum, with most studies either unpublished [2,20], limited to specific practitioner groups [21] or of small sample sizes [22].

There has been a recent call within the wider animal-assisted therapy sector towards greater professionalisation to legitimise this line of work [23]. For this to move forwards within an EAS context, it is imperative that we have an in-depth understanding of these services as they are positioned today. Clarity in this area has the potential to highlight where gaps exist in our knowledge of how EAS is being provided, along with the strengths, weaknesses and challenges faced by practitioners. This information would help to pave the way for solutions that could ultimately strengthen EAS and facilitate legitimisation and growing professionalisation within the sphere of health and wellbeing. With this in mind, the following study aimed to address a lack of knowledge in the area of EAS, with a focus on practitioners’ perspectives. A purpose-designed online survey was developed to assess EAS practitioners’ backgrounds, the nature of the service on offer and their perspectives in regards to both the service they provide and the wider field. It was hoped that the investigation would provide important information on practitioners’ approaches to the field and challenges faced and offer valuable insights into how the sector can best move forwards.

## 2. Materials and Methods

### 2.1. Participants

Practitioners providing any type of Equine-Assisted Service (EAS), e.g., equine-assisted physical therapy, mental health therapy, learning, therapeutic riding, horsemanship, were invited to take part in this study via email and advertisements placed on social media platforms (e.g., Facebook) between June and September 2021. The survey was also shared amongst EAS practitioners and organisations, as well as a number of related print media publications. Completed surveys were screened to ensure the inclusion criteria were met (aged 18+ years, provision of informed consent) and for data quality (i.e., sufficiently completed responses). A total of 145 individuals were removed following screening, leaving a dataset comprising 405 cases. Full details on the participants can be found in Section 3.

### 2.2. Survey

A purpose-designed survey (“Practitioner Experiences and Perspectives of Equine-Assisted Services”, written in English) was developed, which aimed to collect information on practitioners’ backgrounds, experiences and perspectives of EAS. The survey comprised 5 sections:

Section 1 collected basic demographic information including gender (male, female, other, prefer not to say), age (18–29, 30–39, 40–49, 50+ years), highest level of completed education (secondary, tertiary, professional, postgraduate) and professional background (therapy service background (professional service related to health), non-therapy service background (an occupation unrelated to health)). Participants were also required to indicate whether or not they held an equine-related qualification (yes, no), and, if so, the nature of this (open-ended) and the amount of equine-related experience (i.e., any involvement with horses) they had accrued (none, <2 years, 2–5 years, 6–10 years, 10+ years).

Section 2 explored practitioner training. Respondents were required to provide details on their EAS-related qualifications, i.e., those that involved training specific to practice, including qualification provider (open-ended), amount of training undertaken prior to practice (none, 1–5 days, 6–10 days, 11–20 days, block release (>20 days over 10–12 months), 1–3 years, 4 years +, other), independent accreditation (yes, no), number of Continuous Professional Development days per year (0, 1, 2–5, 6–10, 10+) and EAS organisation membership (open-ended). Participants were also asked whether they provided EAS training to other practitioners (yes, no).

Section 3 consisted of questions related to the nature of the EAS offered by the participant. Respondents were initially required to indicate whether they offered a “therapy” or “non-therapy”-related EAS. Therapy services were defined as those that incorporated horses into an already recognised health service, e.g., mental health, speech therapy, occupational therapy, physical therapy. Non-therapy services were defined as those that incorporated horses into their programmes for other purposes, e.g., coaching, experiential learning, education, therapeutic/adaptive riding or horsemanship. For the purpose of analysis, these individual services (both therapy and non-therapy) were subsequently collapsed into those that focused on physical health, mental health, learning and riding. Participants were then asked whether they provided a ground-based-only EAS (i.e., one in which clients observe or interact with horses from the ground only) or a mounted/mixed EAS (i.e., one in which clients are mounted on horses and/or interact with them from the ground). Participants were required to indicate the length of time they had been providing their service (<10 years, >10 years); the type of client they catered for, i.e., the issues that clients presented with (open-ended); the age of their clients (<12, 12–17, 18–24, 25–64, 65+ years); and information regarding the EAS sessions they offered, including duration (<30 min, 30–60 min, 61–90 min, 90+ min), frequency (more than once a week, once a week, once a fortnight, once a month, individually tailored, other), schedule (6-week block, 7–12-week blocks, ongoing, tailored to client, one-off sessions, other) and horse incorporation (same horse each week, different horse each week). A question was also posed in relation to the inclusion of rescue or retired racehorses as part of the equine team (yes, no).

Section 4 was designed to explore participants’ perceived understanding of equine- and service-related issues. Practitioners were initially required to indicate on a 5-point Likert scale (1 = not at all important to 5 = extremely important) how important they considered the quality of the relationship to be between the horse employed in their service and both the client and practitioner. Participants were then required to respond to a series of knowledge-based items designed to assess how well informed they considered themselves to be in relation to both horses and the service they offered, e.g., “ability to read important cues from the horse”, “horse health and welfare”. Responses were made using a 5-point Likert scale, ranging from 1 (low confidence in knowledge level—would like to know more) to 5 (extremely knowledgeable—expert level).

Finally, Section 5 was designed to assess practitioners’ perceptions of challenges in the field of EAS and feelings of work satisfaction. Participants were required to indicate how important they considered a series of statements (e.g., “a clear set of definitions for the different services to reduce confusion”, “equine and client welfare”) in terms of making progress in the field now and the future. Responses were made using a 5-point Likert scale, ranging from 1 (not at all important) to 5 (extremely important).

### 2.3. Procedure

EAS practitioners interested in taking part in the study were directed to the survey hosted on the online platform, Qualtrics. The aims of the survey and terminology used were briefly explained at the outset. For clarity, terminology consistent with Woods et al.’s (2021) optimal terminology in EAS was employed. The survey was anonymous, with no identifying information collected. Following the provision of consent, participants were able to complete the survey, with responses recorded automatically. The survey remained open between June and October 2021.

### 2.4. Data Analysis

Statistical analysis was performed using SPSS version 29. Since this study was largely exploratory in nature, descriptive statistics were initially carried out to determine the demographic profile of the participants, the nature of the EAS they provided and the training accrued. A series of chi-squared tests and ordinal logistic regressions were subsequently conducted to determine the association between these variables and outcome measures of equine- and service-related knowledge, challenges in the field.

## 3. Results

### 3.1. Demographic Information

As can be seen from Table 1, the vast majority of the participants were female, over the age of 50 years and came from the UK/Ireland. Together, most of the sample came from the Global North. Most of the sample held a tertiary-level qualification or above, and the majority of respondents (*n* = 237, 58.5%) reported having an equine-related qualification, the most common being a riding instructor qualification or certificate arising from a short course in equine studies/management/science; 168 respondents (41.5%) held no equine-related qualification. Just over half of the sample indicated that they came from a “non-therapy” background as opposed to a “therapy” background. Individuals who held an equine-related qualification were significantly (*p* < 0.001, Fisher’s Exact test) more likely to come from a non-therapy background (*n* = 162, 68.4%) than a therapy background (*n* = 75, 31.6%). Most of the sample had over 10 years of practical experience working with horses in some capacity.

### 3.2. Practitioner Training

Most of the practitioners who took part in the survey (*n* = 274, 69.0%) reported having accredited training (see Table 2). However, when data were adjusted to include only those organisations that offered independently accredited training, this figure decreased (*n* = 204, 51.3%); nearly half of the sample (*n* = 201, 48.7%) had received no independently accredited training.

A very broad spectrum of training providers was reported by the practitioners, with nearly a third of the sample (*n* = 128, 31.6%) having received training from more than one organisation. The Equine-Assisted Growth and Learning Association (EAGALA) was the most frequently cited training organisation, followed by a US-based body, the Professional Association of Therapeutic Horsemanship (PATH International), and a UK/Ireland-based organisation, the Riding for the Disabled Association (RDA). A large number (>60 organisations) of smaller, more independent training providers were reported by others. A small number (*n* = 50, 8.4%) of practitioners reported that they did not receive training from an EAS organisation prior to practice (Table 2).

The nature of the training undertaken by the practitioners varied widely (Table 3), with a mixture of accredited and non-accredited training providers cited. The most common type of training (29.7%) comprised block release, consisting of at least 20 days of training over a 10–12-month period. Over a third (37.4%) of the sample had received less than 20 days of training, whilst an additional 5% had received no training. Most participants (*n* = 351, 86.9%) had undertaken some form of Continuous Professional Development (CPD), with most engaging in 2–5 days of CPD per year. Just under half of the respondents (*n* = 184, 46.5%) reported undertaking advanced-level training in EAS.

### 3.3. Nature of EAS

Most participants had offered their service for over 6 years and saw 10 or fewer clients per week. Just under half of the practitioners offered a ground-based-only service; most adopted a blended approach to EAS, combining ground and mounted work (Table 4).

**Figure 1:**
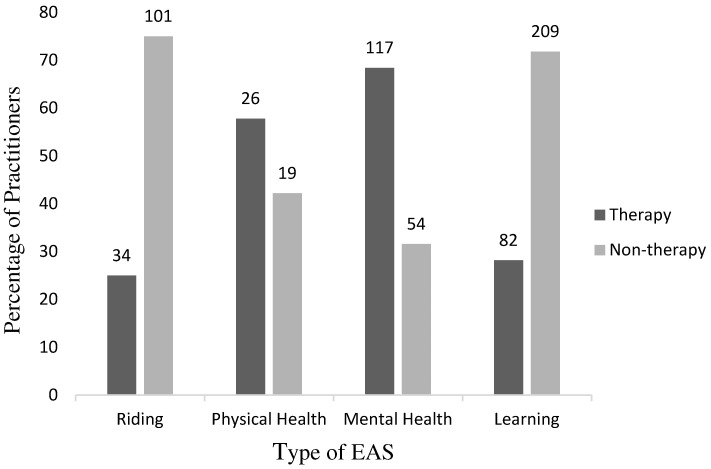
The number and percentage of practitioners from therapy and non-therapy backgrounds offering each type of EAS.

A wide range of EA services was provided, with the most common being learning-related, i.e., with a focus on improving cognitive functioning; nearly half of the sample (*n* = 187, 46.4%) offered more than one type of EAS. Only a small number of the participants ran a service aimed at improving their clients’ physical health. The type of EAS provided was related to the practitioners’ background experiences. Significantly more practitioners who provided a mental health service came from a therapy background as opposed to a non-therapy background (*p* < 0.001, binomial test) (Figure 1). By contrast, practitioners who offered an EAS focused on learning or riding were significantly more likely to come from a non-therapy background than a therapy background (*p* < 0.001, binomial tests). The provision of an EAS that focused on improving clients’ physical health was not found to be significantly related to the practitioners’ background experience (*p* > 0.05, binomial test).

**Figure 2:**
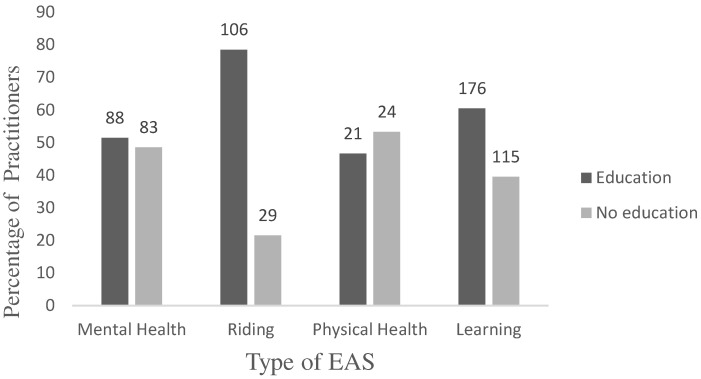
The number and percentage of practitioners offering each type of EAS according to equine-related educational background.

The type of EAS offered was also related to whether or not practitioners held an equine-related qualification, with more of those individuals who ran a learning or riding service having a qualification in this area than would be expected by chance alone (*p* < 0.001, binomial tests) (Figure 2).

Most of the EA services operated weekly, were up to an hour in length and often employed the same horses from session to session (Table 5). The typical schedule of sessions was mixed, with most practitioners either offering a block of sessions or tailoring the programme to the needs of the client. Almost 40% of participants also provided practitioner training as part of their business. Rescue horses were relatively common, incorporated by over half of the participants; a smaller number of respondents reported using retired racehorses in their service.

**Figure 3:**
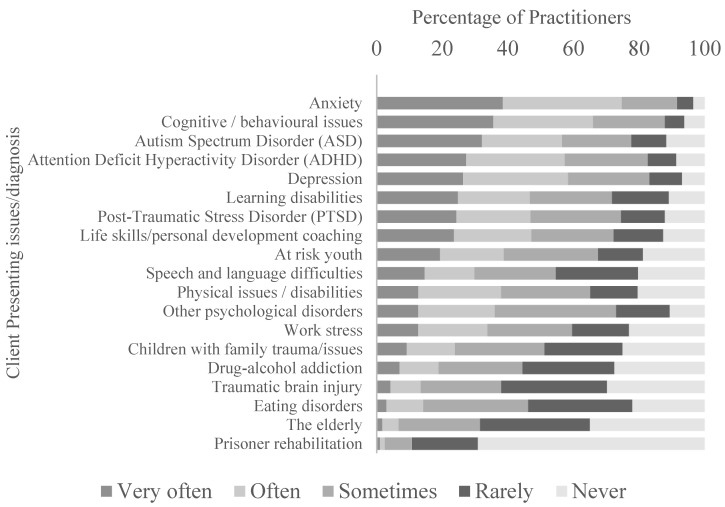
The frequency of occurrence of presenting issues reported by EAS practitioners.

The clients taken on by EAS practitioners presented with a broad spectrum of health and lifestyle issues, the most widespread being anxiety and cognitive/behavioural problems (Figure 3). A large number of clients had also been diagnosed with conditions including Autism Spectrum Disorder (ASD), Attention Deficit Hyperactivity Disorder (ADHD) and depression.

### 3.4. Equine- and Service-Related Knowledge

The mean scores for each of the knowledge-based items included in the survey can be seen in Table 6. Perceived knowledge scores were highest for items relating to interpreting behavioural cues from the horse and health and safety, and lowest in relation to areas including current research in the fields of EAS, equine science and horsemanship.

A series of ordinal logistic regression analyses with proportional odds were conducted to investigate whether any of the independent variables under focus in this study (equine-related education (yes or no), type of EAS (ground-based-only or mounted/mixed service), practitioner background (therapy or non-therapy) and EAS experience (less than/more than 5 years of experience) served as predictors for any of the knowledge-based item scores.

The model was found to be a good fit for all of the regression analyses (see Table 7), with the assumption of proportional odds met, as assessed by a full likelihood ratio test for all items. Equine education served as a significant predictor for three of the knowledge-based items (horse body language, horse health and welfare and the broad field of equine science). Practitioners who had a qualification in an equine-related field scored higher for all of these items than those who did not. The type of EAS was found to be a significant predictor of practitioners’ knowledge scores for five of the items, namely, the ability to read important cues from the horse, the ability to read important cues from the client, horse body language, health and safety and current research in the field. Practitioners who offered a ground-based-only service scored higher for these items than those who provided a mounted/mixed service. Practitioner background significantly predicted knowledge-based scores for the ability to read cues from the client and current research in the field, with participants from a therapy background scoring higher for these items than those from a non-therapy background. Finally, practitioners who had more than 5 years of EAS experience had significantly higher odds of achieving higher scores for all knowledge-based items on the survey than those with less experience.

### 3.5. Challenges in the Field of EAS

**Figure 4:**
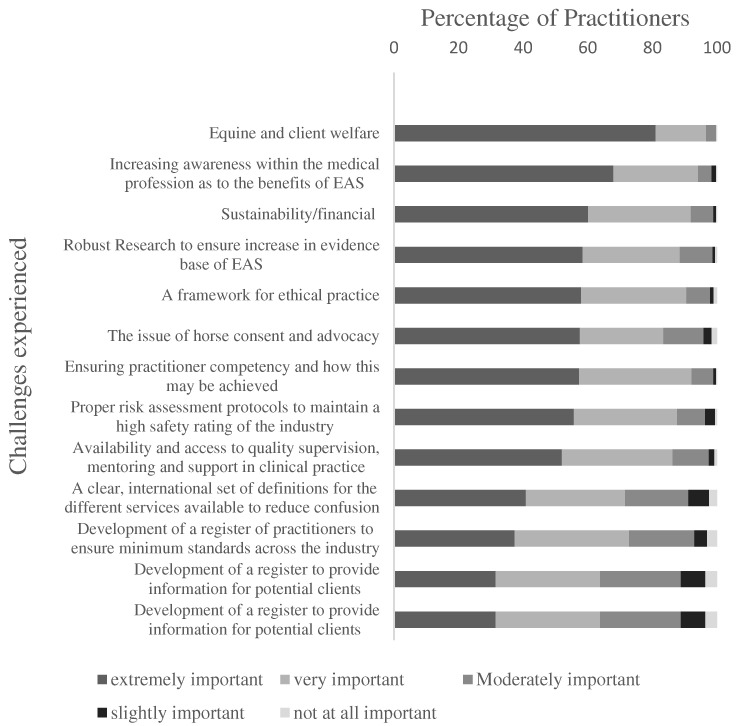
The degree of importance that EAS practitioners attached to each of the challenges presented on the survey.

Mean scores for practitioner-perceived challenges in the field of EAS are outlined in Table 8, with the degree of importance illustrated in Figure 4. Scores were highest for five items related to equine and client welfare, increasing awareness amongst the medical profession and issues around sustainability or financial viability, and were lowest for items related to the development of a register and the establishment of a governing body.

A series of ordinal logistic regression analyses with proportional odds were conducted to examine if some of the key independent variables that might influence practitioners’ perceived challenges, such as the type of EAS (ground-based-only or mounted/mixed service), practitioner background (therapy or non-therapy), EAS experience (less than/more than 5 years of experience) or EAS training (less than/more than 20 days training), served as predictors for any of the practitioner-perceived challenge scores.

The model was found to be a good fit for four of the practitioner-perceived challenge items (see Table 9), with the assumption of proportional odds met, as assessed by a full likelihood ratio test for these items. Practitioner background was a significant predictor for three of the challenge items (a clear set of definitions to reduce confusion, the need for a governing body to establish minimum standards and the availability and access to quality supervision/mentoring). Practitioners who came from a therapy background scored the importance of these items significantly higher than those who came from a non-therapy background. The type of EAS was also a significant predictor for two challenge items (a clear set of definitions to reduce confusion and the need for a governing body to establish minimum standards), with practitioners who provided a mixed/mounted service rating these challenges to be of higher importance than those from a ground-based-only service. In addition, the amount of EAS training undertaken by practitioners was a significant predictor for four of these items, specifically, a clear set of definitions to reduce confusion, the need for a governing body to establish minimum standards, the availability and access to quality supervision/mentoring and a framework for ethical practice. Practitioners who had more than 20 days of EAS training scored significantly higher than those who had less than 20 days of training.

## 4. Discussion

Although some previous studies have been conducted in regards to specific EAS practitioner groups [2,20,21,22,24], this is the first published study of its kind to explore the perspectives of practitioners across the spectrum of Equine-Assisted Services.

### 4.1. Practitioner Characteristics

This study provides interesting insights into the type of people who practice EAS. The vast majority of respondent practitioners were female and over 50 years of age. This profile concurs with other studies in the area [21,24]. One might have expected a slightly younger age profile given the relatively recent emergence of EAS as a career choice. Moreover, online surveys typically attract a younger audience [25]. It can be challenging to get started in the field of EAS, whether because of problems finding training and/or peer groups. It may be that the survey was not available to younger practitioners just starting out in their field and not yet part of a peer community [26]. That said, most of the practitioners surveyed had been educated to at least tertiary level; this points to EAS perhaps serving as a second career, whether for those working in the health and wellbeing sector or for individuals already working in the equestrian field. It must be noted that most of the sample came from the Global North, possibly because the survey was written in English; the potential limitations of this are considered later.

### 4.2. Practitioner Training

Incorporating horses into a human health service involves a unique and complex knowledge and skill set for practitioners, including specific service and people skills, disability awareness, equine knowledge (including an appreciation of equine agency) and effective horse–human interaction management, all within the greater context of wellbeing. It is essential that these skills are well developed, as interacting with horses, whether mounted or on the ground, involves a significantly increased risk of injury [27,28]. This aspect is rarely discussed in EAS research [29], despite the fact that the client base is often vulnerable (e.g., children or those with learning difficulties), with explicit consent sometimes difficult to obtain. The skills required to meet these safety obligations are predominantly equine-related. Somewhat worryingly, the results of this study point to a relatively high number of practitioners (41.5%) holding little, or no, equine-related education. This is of particular concern given the significant number of rescue and retired racehorses incorporated by practitioners in this study; these animals, by their very nature, often require time and specialist support during the transition to EAS or other work [30]. Many of these horses will be subject to considerable changes, including a new environment, management practices and uncertainty, as to what will be expected of them. Some individuals will have experienced repeated rehoming and changes to handlers or personnel. Others may have behavioural or physical issues that need to be addressed. These factors alone can present real challenges, even during the initial settling period. Practitioners working in the mental and physical health domains of EAS were particularly likely to have little, or no, equine-related education, perhaps indicating a therapy, as opposed to an equine, focus in regards to their training. Since a relatively large proportion of participants (40%) in this study also provided EAS training as part of their service, the shortcomings in practitioner knowledge outlined lend additional cause for concern. It may be argued that most of the practitioners surveyed had 10 years or more of equine-related experience, which may compensate somewhat for deficiencies in equine-specific qualifications. However, several studies have failed to show a correlation between equine or riding experience and the ability to correctly identify equine behaviours [31] or appraise horse welfare [32,33], areas known to be closely linked to human safety [34].

Increasing training time alone may not be sufficient to improve equine- or service-related knowledge. For example, a study by Rudd et al. (2022) [31] found that a higher overall number of training sessions in volunteers registered in EAS centres did not equate to better equine behaviour identification; the inclusion of equine-related health training, however, did. It may be that equine health education, which encompasses teachings on normal healthy horse behaviour, alongside the behavioural indicators of disease, pain and stress in a domestic milieu, provides more in-depth and relevant learning, improving positive and negative behaviour recognition in an EAS setting. On the contrary, out-of-context training and hands-on experience (i.e., directed at horse sports, where the goal is maximising performance) may be at odds with the requirements and ethos of the relational aspects of EAS. This area requires further exploration.

This study uncovered considerable variability with regards to the type of training undertaken by practitioners, including the organisations that provided the training and the number of training days acquired to practice EAS. Although there was a relatively high incidence of practitioners who had received at least 20 days of training (44%), some (18%) purported to have less than 10 days, whilst a small number (5%) received none at all, indicating variability in the depth and breadth of practitioner training and education. It seems unlikely that the amount of EAS training levels reported by practitioners is sufficient to cover the broad range of topics outlined, meaning many practitioners may be coming to this field with significant gaps in knowledge that could potentially impact client and horse welfare. It could be argued that the results from this study point to a mistrust or reduced value placed on science or evidence-based knowledge within the sector, with practitioners relying more on knowledge gained through practice. In addition, there may be some uncertainty in regards to the value or relevance of more traditional education or knowledge transfer modes (certificates, further education and supervision) for this very hands-on type of work.

### 4.3. A Move towards Professionalisation and Standardisation

In addition to the “short course” nature of a considerable number of EAS trainings, about half of practitioners surveyed here took part in independently accredited training programmes, suggesting that a vacuum may exist in regards to quality recognised educational standards for those seeking, providing or wishing to fund such services. Commonly offered non-accredited “certificates of completion” can be problematic in any sphere, as they provide little information in regards to attendee competency, further calling into question practitioners’ specialist knowledge [2]. As there is not yet a defined “book of knowledge” or consensus in the field as to the core competencies and standards required to practice EAS [35,36], even accredited courses may not provide the skills, knowledge and competencies necessary to prepare practitioners adequately. In addition, practitioners and training providers must be able to accurately assess whether any prior training or experience aligns with, or is sufficient for, EAS entry requirements. An apparent mismatch between the required and actual experience may be reflected in the anecdotal reports that many who attain training in EAS fail to progress to practice. This study’s findings of high voluntary CPD attendance (40% >6 days/year) indicate that many practitioners are actively seeking knowledge to grow or maintain their skill set. The provision of high-quality evidence-based experiential workshops, an effective educational approach perceived to be beneficial and well received by practitioners [21], as well as more traditional evidence-based CPD approaches, may enhance and fill some of the knowledge or curriculum gaps that currently exist for some practitioners. It is imperative that core competencies in EAS are developed within the context of wellbeing and a One Health–One Welfare framework for all involved, ensuring consistency with EAS requirements and ethos. Consistency in this area would be a step towards professionalisation, a position cited as important for progress and legitimacy within the wider animal-assisted service community [37].

### 4.4. EAS Practice Characteristics

Most of the practitioners offered “learning”-related services, i.e., EAS practices that were designed to improve the cognitive functioning of their clients. This makes sense, considering the fact that over 90% of practitioners reported having clients presenting with anxiety-related health problems. Nearly half of the practitioners, however, offered a service aimed at enhancing several aspects of wellbeing, e.g., physical health and mental health. Again, this is understandable when one considers the broad spectrum of client-based health issues reported here, which can include clients with multiple presenting difficulties. It also makes sense from a business perspective. Delivering a variety of services increases the number of potential clients, as well as providing an additional source of revenue during seasonal declines in demand. The high frequency of anxiety as a reported issue perhaps explains the greater emphasis on learning-based strategies; however, many clients had a range of physical and mental health problems, possibly necessitating a wider focus than learning-related services alone.

As reported by others [20,24,38], EAS sessions were typically weekly and between 30–60 min in length. The number of sessions provided varied quite a bit between practitioners, with a range of delivery options (e.g., one-off sessions, blocks of 5–12 sessions and ongoing provision) reported by participants. This variability in programme delivery may reflect practitioners’ personal preferences, and/or client, referral or funding practices. Some organisations, such as the Riding for the Disabled Association, provide a funded, mostly ongoing provision. Other services, however, including equine-assisted physiotherapy, occupational therapy or psychotherapy, are more expensive and less likely to receive outside funding. Corporate-focused organisations, by contrast, typically deliver day/half-day sessions to suit workplace dynamics. Variability in the number of sessions offered may, therefore, reflect the broad spread of different services being offered by the practitioners.

### 4.5. Horses Employed in EAS

The practitioners sampled here typically selected horses for the client, although a small number gave a choice to the client or the horse. The same horse/s were often partnered with clients over a number of sessions, a practice that ties in with the view that the main benefits of EAS are related to the development of bonds with an animal over time [39,40]. This is also reiterated through the high importance practitioners placed on the client–horse and the client–practitioner relationship. This study is the first of its kind to document the apparently widespread practice of incorporating rescue or retired racehorses into EAS teams; although there may be some issues related to this (see above), it does highlight the positive role that the EAS sector may play in helping to reduce “wastage” in the wider equine industry.

### 4.6. Practitioner-Perceived Knowledge

The practitioners’ perceived knowledge of the equine- and EAS-related topics presented to them in this study was found to be relatively high. Whilst this is a positive finding, it does not necessarily infer actual understanding, as overconfidence has been encountered in equestrians [32,41], as well as in individuals working in wellbeing [42] and other fields (for a review, see [43]). Interestingly, the key areas of knowledge, including awareness of abnormal horse behaviour [33,44], nutritional and health knowledge [45,46], equine emotional state recognition [47,48] and the implementation of good welfare practices [49,50], have been found to be lacking within the equestrian field. The provision of appropriate, high-quality equine training or information can mitigate against this [41,44], although some barriers exist, particularly in relation to the overexposure effects of working within less-than-ideal welfare environments [51].

Practitioners’ perceived knowledge of the equine- and EAS-related issues in this study was found to be significantly related to their background. Individuals who had equine-related training had higher knowledge-based scores for several of the items, including an understanding of horse body language, horse health and welfare and the broader field of equine science. This is understandable, as most of these areas would be taught in any equine-related training programme. Interestingly, differences in perceived knowledge were found between people who offered ground-based-only vs. mixed/mounted EAS, with the people offering the former having higher perceived knowledge scores for five of the knowledge items. As ground-based services can include practitioners from therapy and non-therapy backgrounds, the significance of this is difficult to determine and requires further exploration. Therapy service providers, more predictably, had higher perceived knowledge scores in regards to client cues and research, areas consistent with training in therapy fields. Although the differences found here mirror the diversity in the field, they also hint at weaknesses in the overall knowledge base, especially with regards to equine education level and client issues, again suggesting the need for more comprehensive training programmes that incorporate all areas of EAS.

### 4.7. Practitioner-Perceived Challenges

In light of the shortcomings in the equine-related education and training outlined above, it is somewhat telling that the practitioners themselves overwhelmingly regarded equine and client welfare very highly in terms of challenges faced by those working in EAS. Awareness or recognition of the field by medical professionals was also seen as a particularly important challenge for most, indicating that difficulties may exist with regards to referral from health professionals or social prescribing. Sustainability and financial viability were also seen as important challenges, understandably so, due to the high costs of keeping horses. The challenges regarded as less important were found to be those most related to the regulation or development of a register. This is surprising considering the increasing calls to professionalise and regulate the greater animal-assisted sector and the current situation within the wider traditional and complementary medicine professions, where regulation is now gaining widespread support [52]. However, the fact that regulatory issues were seen to be of greater importance to those from a therapy background in this study is worthy of note. Such individuals are perhaps more familiar with the benefits and protections that can be gained from formal registration, regulation and uniform terminology, along with the public confidence that this can generate. Access to mentoring or supervision was also rated higher for this group, possibly due to the existing compulsory requirements for therapy services as well as the culture of mentoring that exists within these spheres. EAS training also influenced practitioners’ ratings of regulatory and supervision/mentoring challenges, with those having more than 20 days of training rating these challenges of higher importance. Without a clear understanding or knowledge in regards to the benefits that these practices may bring, it would be difficult to view them as important in terms of challenges, a kind of Dunning–Kruger effect or “you don’t know what you don’t know”. On the other hand, greater knowledge with regards to regulatory issues and practitioner support, a subject part of some EAS programmes, would allow greater appreciation of the benefits these might bring.

It is of note that practitioners rated the challenge of horse consent and advocacy as less important than some of the other issues (e.g., robust research). This raises questions in regards to practitioner awareness regarding these issues. It may be that practitioners have yet to fully grasp the significance of consent and advocacy for the safety and wellbeing of clients and horses within EAS and a One Health framework. More education may be required in order to ensure practitioners recognise and include consent and advocacy as an important part of delivery in EAS.

### 4.8. Study Limitations

Certain limitations must be acknowledged in relation to this study. Participants were recruited via email and EAS-related social media platforms. This recruitment approach may have produced a bias towards practitioners who are familiar with these social media platforms, have a significant internet presence or regularly engage online. It may also have excluded some practitioners starting out on their journey who have not yet gained access to peer groups online. In addition, it may be that some participants had a particular motivation for taking part in this type of research, e.g., for the promotion of a certain methodology, training approach, concern or opinion. However, the considerable diversity of practitioners, their backgrounds, services provided, training and distinct views seen here may have enriched the dataset. Although practitioner backgrounds were diverse, a bias towards the Global North was evident within this sample and must be acknowledged. Further work may be required to explore how generalizable the findings are and to investigate whether responses to any of the issues examined vary according to geographic location (not examined here statistically). The time required to complete the survey (~20 min) could have discouraged some practitioners from taking part or finishing. Some of these limitations may be improved in the future by using shorter, more topic-specific questionnaires publicised at a general level (equine industry, health and education sectors), as well as to an EAS-specific audience to further explore the findings uncovered here. Indeed, interview work is currently underway to explore, using a more qualitative approach, some of the challenges faced by EAS practitioners.

## 5. Conclusions

This study provides the first industry-wide look at how EAS is practised, as well as insights into the diverse range of training, education and challenges faced by practitioners. It is clear from the findings that work needs to be conducted to help practitioners deliver the best service to clients through appropriate, in-depth and evidence-based training. In particular, solid foundations in equine education, especially equine health, welfare, behaviour, safety and ethical management, are needed to ensure client and horse safety and wellbeing. In addition, work is needed to ensure that practitioners are well equipped to provide quality services to clients, whatever this may be. This education must be provided and taught within the context of a wellbeing service, in order to avoid the promotion of wellbeing- or welfare-limiting practices; in essence, this should be within a One Health framework. The development of industry-wide core competencies is urgently needed. This step would enable those already practicing EAS to seek out CPD in areas that may be deficient as well as provide a framework for training providers going forward. A move towards professionalisation and consensus within EAS would also signal to funders and policy makers that incorporating horses into health and wellbeing services is a viable option.

## Figures and Tables

**Table 1:** Number and percentage of EAS providers according to demographic background.

Demographic Background	*N*	%
Gender		
Female	381	94.1
Male	21	5.2
Prefer not to say	3	0.7
Age (in years)		
18–29	17	4.2
30–39	57	14.1
40–49	90	22.2
50+	241	59.5
Region of residence		
UK and Ireland	167	41.2
Americas	157	38.8
Oceania	39	9.6
Rest of the world	42	10.4
Horse experience (in years)		
<2	12	3.0
2–5	26	6.4
6–10	32	7.9
>10	335	82.7
Highest completed level of education		
Secondary level	46	11.4
Tertiary level (college/university)	119	29.4
Professional qualification	99	24.4
Postgraduate (masters/PhD)	141	34.8
Equine qualifications		
Qualified instructor	121	33.7 *
Short course in equine studies	90	25.1 *
Diploma/certificate in equine studies	58	16.2 *
Degree/postgraduate in equine studies	31	8.6 *
British Horse Society Stage 2	28	7.8 *
Other	30	8.3 *
Nature of previous experience		
Therapy background	164	40.5
Non-therapy background	241	59.5

* % out of total number of education items chosen (*n* = 358) by 236 practitioners who had equine education, participants could choose more than 1 option, 41.5% (168) of the sample chose none of these options.

**Table 2:** The number and percentage of participants who had received training prior to practice along with overall accreditation status.

Training	*N*	%
Training Organisation		
EAGALA	108	18.1
PATH	73	12.2
RDA	42	7.0
Festina Lente	27	4.5
Eponaquest	24	4.0
LEAP	20	3.3
Equine Psychotherapy Institute	19	3.2
IFEEL	15	2.5
Natural Lifemanship	14	2.3
EAHAE	14	2.3
Other	192	32.1
None	50	8.4
Training Accredited *		
Yes	274	69.0
No	123	31.0
Adjusted to independent accreditation.		
Yes	204	51.3
No	194	48.7

* Note that this figure includes accreditation that is not independent of training provider. Some providers deliver training that has no accreditation.

**Table 3:** Number and percentage of participants according to specifics of EAS training.

Training Specifics	*N*	%
Length of training		
No training	20	5
1–5 days	42	10.4
6–10 days	31	7.7
11–20 days	78	19.3
Block release (>20 days over 10–12 months)	120	29.7
1–3 years (certificate/diploma/degree)	46	11.3
4 years (Post-graduate training)	12	3.0
Other	55	13.6
Continuous Professional Development (days/year)		
None	53	13.1
1	43	10.6
2–5	137	33.9
6–10	79	19.6
>10	92	22.8
Have you received any advanced training in EAS?		
Yes	184	46.5
No	212	53.5

**Table 4:** The number and percentage of participants according to the nature of the EAS.

Nature of Service	*N*	%
Category of service		
Learning	291	72.2
Mental health	171	42.4
Riding	135	33.5
Physical health	45	11.2
Primary nature of service		
Ground-based-only	202	49.9
Mounted/mixed	203	50.1
Length of time providing EAS (in years)		
<1	29	7.2
1–2	60	14.8
3–5	84	20.7
6–10	110	27.2
>10	122	30.1
Client age		
Children (<12 years)	114	28.1 *
Adolescents (12–17 years)	128	31.6 *
Adults	246	60.7 *
All ages	219	51.6 *
Format of sessions		
Group sessions only	69	17.0
Individual sessions only	160	39.5
Group and individual sessions	163	40.2
Other	13	3.2
Clients (per week)		
<5	135	33.6
6–10	110	27.4
11–20	81	20.1
21+	76	18.9

* Participants could choose more than 1 category.

**Table 5:** Practice patterns and views regarding horses, selection and service specifics.

The Service	*N*	%
Selection of horse		
Horse selected by practitioner	127	33.4
Horse selected by client	94	24.7
Client selected by horse	64	16.8
Other	95	25.0
Session to session horse selection		
Same horses	246	61.2
Different horses	156	38.8
EAS horse background		
Rescue horses as part of the team	255	58.9
Retired racehorses as part of the team	115	30.0
Importance of relationships (very/extremely important)		
Horse–Practitioner	337	88.0
Horse–Client	313	81.7
Length of session		
<30 min	14	3.5
30–60 min	224	55.7
61–90 min	114	28.4
>90 min	50	12.4
Frequency of sessions		
>Once per week	12	2.5
Weekly	271	56.5
Fortnightly	45	9.4
Monthly	22	4.6
Individually tailored to client	111	23.1
Other	19	4.0
Schedule of service		
6-week blocks	69	12.6
7–12-week blocks	76	13.9
Ongoing	119	21.8
Varies according to client	212	38.8
One-off sessions	49	9.0
Other	21	3.8
Practitioner training provided		
Yes	159	39.4
No	245	60.6

**Table 6:** Practitioners’ mean +/− SD scores for each of the knowledge-based items included in the survey.

Item	Mean	SD
Ability to read important cues from the horse	3.59	1.23
Health and safety	3.51	1.10
Choose appropriate horse for therapy/client	3.47	1.16
Ability to read important cues from the client	3.47	1.20
Horse body language	3.43	1.23
Horse health and welfare	3.33	1.26
Horse behaviour in relation to pain/discomfort	3.20	1.31
Broad field of horsemanship	2.95	1.22
Current research in the field	2.78	1.24
Broad field of equine science	2.62	1.21

**Table 7:** Predictors and results in relation to perceived equine- and service-related knowledge items.

Predictor Variables	Wald Chi-Square	Exp(B)	Lower 95% CI	Upper 95% CI	*p*
**Ability to read important cues from the horse** (Pearson goodness-of-fit X^2^ (56) = 71.211, *p* = 0.086)					
Type of EAS	4.219	0.680	0.471	0.983	0.040
EAS experience	7.685	0.595	0.412	0.859	0.006
**Health and safety** (Pearson goodness-of-fit X^2^ (56) = 61.526, *p* = 0.285)					
EAS experience	5.248	0.648	0.447	0.939	0.022
**Choose appropriate horse for therapy/client** (Pearson goodness-of-fit X^2^ (56) = 60.436, *p* = 0.319)					
EAS experience	20.357	0.421	0.289	0.613	<0.001
**Ability to read important cues from the client** (Pearson goodness-of-fit X^2^ (56) = 68.359, *p* = 0.124)					
Type of EAS	10.215	0.546	0.377	0.791	0.001
Background	14.276	2.070	1.419	3.020	<0.001
EAS experience	9.321	0.563	0.389	0.814	0.002
**Horse body language** (Pearson goodness-of-fit X^2^ (56) = 40.038, *p* = 0.947)					
Equine education	4.222	1.485	1.018	2.167	0.040
Type of EAS	4.770	0.662	0.458	0.959	0.029
EAS experience	6.741	0.613	0.424	0.887	0.009
**Horse health and welfare** (Pearson goodness-of-fit X^2^ (56) = 55.435, *p* = 0.496)					
Equine education	7.946	1.713	1.178	2.490	0.005
Type of EAS	4.873	0.663	0.460	0.955	0.027
EAS experience	6.776	0.616	0.428	0.887	0.009
**Horse behaviour in relation to pain/discomfort** (Pearson goodness-of-fit X^2^ (56) = 52.077, *p* = 0.624)					
EAS experience	4.895	0.663	0.460	0.954	0.027
**Broad field of horsemanship** (Pearson goodness-of-fit X^2^ (56) = 53.690, *p* = 0.563)					
EAS experience	12.633	0.515	0.357	0.743	<0.001
**Current research in the field** (Pearson goodness-of-fit X^2^ (56) = 46.790, *p* = 0.805)					
Type of EAS	8.008	0.589	0.408	0.850	0.005
Background	8.650	1.743	1.204	2.525	0.003
EAS experience	13.789	0.499	0.346	0.720	<0.001
**Broad field of equine science** (Pearson goodness-of-fit X^2^ (56) = 49.287, *p* = 0.725)					

Only significant results are included.

**Table 8:** Practitioners’ mean +/− SD perceived scores for perceived challenges in the field.

Item	Mean	SD
Equine and client welfare	4.77	0.52
Increasing awareness within the medical profession	4.60	0.67
Sustainability/financial viability	4.51	0.69
Ensuring practitioner competency	4.48	0.69
A framework for ethical practice	4.45	0.77
More robust research	4.45	0.75
Proper risk assessment protocols	4.39	0.82
The issue of horse consent and advocacy	4.35	0.91
Availability and access to quality supervision/mentoring	4.35	0.81
A clear set of definitions to reduce confusion	4.01	1.04
Development of a register to ensure minimum standards	4.00	1.01
Development of a register to provide information clients	3.80	1.08
Governing body to establish minimum standards	3.63	1.18

**Table 9:** Predictors and results in relation to perceived practitioner challenges.

Predictor Variables	Wald Chi-Square	Exp(B)	Lower 95% CI	Upper 95% CI	*p*
**Clear set of definitions to reduce confusion** (Pearson goodness-of-fit X^2^(56) = 56.435, *p* = 0.459)					
Background	4.023	1.501	1.009	2.234	0.045
EAS experience	9.734	1.872	1.262	2.775	0.002
EAS training	10.307	0.530	0.360	0.781	0.001
**Governing body to establish minimum standards** (Pearson goodness-of-fit X^2^(56) = 61.473, *p* = 0.286)					
Background	5.577	1.596	1.083	2.352	<0.001
Type of EAS	5.802	1.602	1.092	2.352	0.016
EAS training	12.155	0.509	0.348	0.744	0.000
**Availability and access to quality supervision/mentoring** (Pearson goodness-of-fit X^2^(56) = 52.988, *p* = 0.590)					
Background	9.325	1.942	1.268	2.975	0.002
EAS training	13.983	0.457	0.303	0.689	<0.001
**A framework for ethical practice** (Pearson goodness-of-fit X^2^ (56) = 41.831, *p* = 0.920)					
EAS training	4.775	0.629	0.415	0.593	0.029

Only significant results are included.

## Data Availability

Data are contained within the article.
